# ID 148 - Terapia de Estimulação Cognitiva (CST) para o Tratamento de Pessoas com Doença de Alzheimer Leve e Moderada: análises de custo-utilidade e impacto orçamentário

**DOI:** 10.5327/2237-9622.2025.v34s1.148

**Published:** 2025-11-25

**Authors:** Layssa Andrade Oliveira, Juliana Yukari Viscondi, Tassiane Cristine Santos de Paula, Haliton Alves Oliveira, Rosa Camila Lucchetta

**Keywords:** terapia de estimulação cognitiva, doença de Alzheimer, análise de custo-efetividade

## Abstract

**Introdução:**

A doença de Alzheimer (DA) é a principal causa de demência em idosos, caracterizada pelo comprometimento de diversos domínios cognitivos, como memória, julgamento e confusão. Embora os tratamentos medicamentosos sejam amplamente utilizados, a terapia de estimulação cognitiva (CST) emergiu como uma intervenção não medicamentosa promissora. No Brasil, a CST foi adaptada e validada, mas sua aplicação ainda é limitada. A CST envolve exercícios cognitivos estruturados e repetitivos oferecidos em grupo e conduzida por facilitadores treinados, consistindo em 14 encontros de 45 minutos cada sessão, sendo realizada em até duas vezes por semana. Dessa forma, este estudo tem como objetivo avaliar a custo-utilidade e o impacto orçamentário (IO) da CST para o tratamento de pessoas com DA leve à moderada, para o Sistema Único de Saúde (SUS).

**Método:**

Foi realizada uma avaliação econômica (AE) para estimar a razão de custo-efetividade incremental (RCEI) da CST em grupo combinada ao tratamento padrão (intervenção), em comparação ao tratamento padrão isolado (comparador). Utilizou-se um modelo de Markov com horizonte temporal de um ano, desfecho de ano de vida ajustado pela qualidade (QALY) e custos com treinamento e equipamentos. Para a estimativa do IO, foi considerada uma taxa de difusão da intervenção de 20% a 100%, em cinco anos.

**Resultados:**

A intervenção resultou em um ganho de 0,0041 QALY com um aumento de custo de R$ 152,42, gerando uma RCEI de R$ 37.280,24/QALY ganho, considerada custo-efetiva em relação ao limiar de disposição a pagar estabelecido pela Conitec (42,2% de probabilidade de ser custo-efetiva). Considerando uma população elegível variando de 123.377 a 160.452 a incorporação da CST geraria impacto orçamentário de R$ 3,26 milhões no primeiro ano, chegando a R$ 5,41 milhões em cinco anos, com um total acumulado de aproximadamente R$ 17,2 milhões em cinco anos, podendo chegar a R$ 29 milhões, conforme variações no número de grupos e pacientes atendidos nas análises de sensibilidade.

**Conclusão:**

A análise de custo-utilidade indicou que a intervenção investigada oferece benefício clínico maior e aumento de custo total em comparação ao tratamento padrão, sendo considerada custo-efetiva dentro do limiar adotado pelo SUS. A análise de impacto orçamentário sugere incremento acumulado em cinco anos, dependendo do, do número de grupos atendidos por ano e do número de pacientes por sessão.

